# Pain Assessment Disparities by Race, Ethnicity, and Language in Adult Hospitalized Patients

**DOI:** 10.1016/j.pmn.2023.03.012

**Published:** 2023-05-03

**Authors:** Aksharananda Rambachan, Hamedullah Noorulhuda, Margaret C. Fang, Marilyn Bazinski, Solmaz Manuel, Colin Hubbard, Priya Prasad

**Affiliations:** * Division of Hospital Medicine, University of California, San Francisco, California; † School of Medicine, University of California, San Francisco, California; ‡ School of Nursing, University of California, San Francisco, California; § Department of Anesthesia and Perioperative Care, University of California, San Francisco, California

## Abstract

**Aim:**

Nurses assess patients’ pain using several validated tools. It is not known what disparities exist in pain assessment for medicine inpatients. Our purpose was to measure differences in pain assessment across patient characteristics, including race, ethnicity, and language status.

**Methods:**

Retrospective cohort study of adult general medicine inpatients from 2013 to 2021. The primary exposures were race/ethnicity and limited English proficiency (LEP) status. The primary outcomes were 1) the type and odds of which pain assessment tool nursing used and 2) the relationship between pain assessments and daily opioid administration.

**Results:**

Of 51,602 patient hospitalizations, 46.1% were white, 17.4% Black, 16.5% Asian, and 13.2% Latino. 13.2% of patients had LEP. The most common pain assessment tool was the Numeric Rating Scale (68.1%), followed by the Verbal Descriptor Scale (23.7%). Asian patients and patients with LEP were less likely to have their pain documented numerically. In multivariable logistic regression, patients with LEP (OR 0.61, 95% CI 0.58–0.65) and Asian patients (OR 0.74, 95% CI 0.70–0.78) had the lowest odds of numeric ratings. Latino, Multi-Racial, and patients classified as Other also had lower odds than white patients of numeric ratings. Asian patients and patients with LEP received the fewest daily opioids across all pain assessment categories.

**Conclusions:**

Asian patients and patients with LEP were less likely than other patient groups to have a numeric pain assessment and received the fewest opioids. These inequities may serve as the basis for the development of equitable pain assessment protocols.

Pain is extremely common among hospitalized patients, with more than 50% of adult general medicine inpatients reporting pain while hospitalized (Lin et al., 2021). Inadequate treatment of acute pain is associated with negative short and long-term patient outcomes, including muscle loss, poor sleep, loss of economic productivity, the development of chronic pain, and most importantly, unalleviated suffering (Kobayashi et al., 2021; Rummans et al., 2018).

The 1990’s “pain as the fifth vital sign” campaign led to the greater inclusion of pain related questions in patient satisfaction surveys. Regulatory requirements across hospital systems also focused on pain assessments, documentation, and opioid administration (Macintyre et al., 2020). More recently, clinicians and professional societies have recognized the significant limitations with our current approach to pain assessment and documentation (Lin et al., 2015; Scher et al., 2018). Nursing assessments for pain are separated into “self-reporting tools” and “behavioral tools” (Hadjistavropoulos et al., 2007; Pasero et al., 2016). Self-reporting tools require clinicians to ask patients to “self-report” by choosing the Numeric Rating Scale (NRS), the Verbal Descriptor Scale(VDS), or the FACES Pain Scale-Revised (Pathak et al., 2018). When acute care patients are unable to self-report pain, nurses use the Checklist of Nonverbal Pain Indicators (CNPI) behavioral pain assessment tool (Table 1).

Ideally, behavioral tools are used when a nurse deems that a patient is unable to provide a self-reported assessment (i.e., due to cognitive impairment, inability to communicate, or clinical acuity). Moreover, within self-reporting assessments, nurses are encouraged to utilize the Numeric Rating Scale when possible. The simplicity of a 0–10 scale, minimization of subjective pain terminology, and intended comparability across time have contributed to the Numeric Rating Scale’s ubiquity. Notably, there is movement towards more holistic pain assessments that include functional assessments of a patient, whereby the efficacy of pain treatment is related to the degree of activity achieved (Adeboye et al., 2021; Medico et al., 2017). The Numeric Rating Scale is criticized for its poor validity in non-English speaking, non-white patient populations and variability in what each number (and change across numbers) mean for different patients (Adeboye et al., 2021; Medico et al., 2017; Pathak et al., 2018). Despite these significant limitations, the Numeric Rating Scale remains the standard pain assessment tool for inpatient clinical settings (Safikhani et al., 2017; Williamson & Hoggart, 2005).

In actual clinical care, there may be significant heterogeneity in which pain assessment tools are used, influenced by patient level factors including illness severity and sociodemographic variables, local practices, and clinician biases. The subjective nature of pain and clinician discretion makes pain management susceptible to significant disparities across racial, ethnic, and language-based patient factors (Meints et al., 2019). This study addresses a key gap in the literature by characterizing disparities in pain assessment tools by race, ethnicity, and language. This is important to address, because the differential use of assessment tools and documentation of pain may lead to inequities in medication prescription and administration by clinicians. Despite the most recent guidance, documented pain scores are often a threshold to determine what, if any, dose of pain medications to administer to patients (Pasero et al., 2016; van Dijk et al., 2015). In the inpatient setting, opioid pain medications are commonly used because of the severity of reported pain and the ability for patients to be closely monitored (Donohue et al., 2019).

The purpose of this analysis is twofold. The first aim was to assess for systematic differences among nurses’ documentation of pain assessments across different patient characteristics, including race, ethnicity, and language status. The second aim was then to examine the association between pain assessment type and quantity of opioids administered to hospitalized patients. We chose to focus on opioids given their ubiquity in the inpatient setting and the greater risks associated with inappropriate opioid administration, including risk of long-term opioid dependence (Donohue et al., 2019). These analyses address previously unexplored areas of importance in clinical medicine. The identification of disparities in the use of pain assessment tools and related management specifically by race, ethnicity, and language will enable more equitable approaches to pain management.

## Methods

### Study Population and Data Sources

This was a retrospective cohort study of all adults (age ≥18 years) discharged from the acute care inpatient general medicine service between January 2013 through September 2021 at the University of California, San Francisco (UCSF) Helen Diller Medical Center, a 785-bed urban, academic medical center. All data used in this study were obtained from the hospital’s Epic-based Electronic Health Record (EHR), with specific data elements extracted from Clarity, a relational database that stores Epic data. Clarity contains all patient demographic data, clinical data including vitals, labs, and imaging, and the time-stamped medication administration record. Each patient admission reflected a unique hospitalization, meaning multiple admissions by the same patient would be counted distinctly.

We extracted data on all documented pain assessments performed by nursing. For a data point to be included in this analysis, nurses specified that they (i) completed a pain assessment, (ii) specified the type of pain assessment tool used, and (iii) documented a value for the pain assessment tool. For example, a nurse would document that they are going to use a Pain Assessment Tool (PAT), then select the PAT type as “Numeric Rating Scale” and the value as “8” based on the patient’s response. Nursing pain assessments are performed on admission; after unit transfers; before, during, and after procedures; with routine vital sign checks; and prior to and after analgesic administration. Nurses are trained through onboarding regarding medication safety, administration, and documentation. These data are inputted by nurses into EHR flowsheets. Each data point, including a complete PAT with a patient’s demographic data, represented a row of data in our analysis. We excluded patients who spent a portion of their hospitalization in the Intensive Care Unit. We also excluded incomplete or missing pain assessment values. The UCSF Institutional Review Board for Human Subjects Research approved this study with a waiver of informed consent. The datasets used for the current study are available upon request from the corresponding author.

### Covariate Data Collection

We collected additional data across patient demographics, hospitalization, and medical factors. Demographic variables included age and self-reported gender. Hospitalization and medical variables included year of admission, length of stay, if cancer pain was the patient’s primary hospital problem, whether a patient was prescribed opioids prior to admission, and whether the patient was placed on comfort care during their admission to represent end-of-life care. We also calculated a Elixhauser comorbidity score for each patient (van Walraven et al., 2009). These variables were selected because of their relevance for pain assessment and management in the inpatient setting.

## Primary Outcomes

Two primary outcome categories were analyzed: the frequency and odds of which type of pain assessment tools were used, and the relationship between pain assessment and administered opioid pain medication. The first outcome was the type of pain assessment tool utilized by nursing. Options included self-reported pain tools: Numeric Rating Scale, Verbal Descriptor Scale, FACES pain scale, or behavioral tools, including the Checklist of Nonverbal Pain Indicators (Table 1), or “Other,” which included the use of inappropriate tools (i.e., use of a pediatric assessment on an adult patient), or nurse documentation “unable to assess,” “nonverbal,” “assume pain is present,” or “off the floor ” (meaning not present on their medical unit). We also examined documentation restricted to nurse performed “self-report tools” comparing numeric rating to verbal descriptor/FACES pain scale. Within the cohort of self-report only, using a multivariate logistic regression, we examined the odds of a patient receiving a numeric pain assessment versus another pain assessment tool. The second outcome was the number of opioids associated with each patient assessment type, calculated as the average morphine milligram equivalent (MME) per patient/per day. We examined this outcome across (i) all pain assessment types, including all self-reported and behavioral assessments and (ii) restricted to only self-reported assessments, comparing the Numeric Rating Scale to the Verbal Descriptor Scale/FACES pain scale.

### Primary predictors

Because self-reported pain assessment tools rely on communication between nurses and patients, we hypothesized that there would be differences in the use of self-reported pain tools by language and race/ethnicity. Race/ethnicity was categorized as American Indian/Alaska Native, Asian, Black/African American, Latino, Multi-Race/Ethnicity, Native Hawaiian/Other Pacific Islander, Other, and white. These race/ethnicity groupings are consistent with U.S Census and NIH reporting standards (NIH, 2015). Patients were categorized as Latino if Hispanic was their documented ethnicity, no matter their racial status, consistent with standard practice (Howell, 2017). While we are describing differences across racial and ethnic groups, we recognize that these are socially defined, not genetic, groupings (Kaplan & Bennett, 2003). Limited English proficiency (LEP) status was defined as having a self-identified primary language other than English and intake assessment by the patient reporting that they require an interpreter. Our hospital has 24/7 access to video or telephone medical interpreters.

### Statistical Analysis

All analyses were done using Stata software v.17. Baseline demographic, hospitalization-related, and comorbidity indices were stratified by pain assessment tool with comparisons using chi-squared or ANOVA tests. Multivariable logistic regression included race/ethnicity, LEP status, age, comorbidity index, cancer pain diagnosis, opioids on admission, length of stay, comfort care, and study year (as a proxy for temporal changes in prescribing). We also tested a regression with the interaction included between race/ethnicity and LEP. As the level of analysis was each documented pain assessment, many patients had multiple rows of data included in this analysis. The same MME average was used for each row of data for each individual patient. Cluster-robust variance estimates were used to account for clustering at the patient level using medical record numbers. All models used a significance level of p ≤ .05.

## Results

Our dataset began with 60,192 patient hospitalizations with 3,046,210 documented data points related to pain assessments. After eliminating patients admitted to the Intensive Care Unit and all incomplete or missing pain assessment values, we included 51,602 patient hospitalizations and 1,858,441 patient-level pain assessment values. Our data reflect the wide diversity of patients managed at our institution (Table 2). The patient population across assessments was 46.1% white, 17.4% Black, 16.5% Asian, 13.2% Latino, 2.0% Multiracial, 0.8% Native Hawaiian or Other Pacific Islander, 0.6% American Indian or Alaska Native, and 3.5% Other/Unknown. The majority, 86.8%, were English speaking and 13.2% were patients with LEP. While our main analysis was performed at the patient-assessment level, we also describe the overall demographic distribution of the individual 51,602 patients in Supplemental Table 1.

### Outcome 1: Types of Pain Assessment Tools Used

There was significant variation across racial/ethnic groups and language status in the types of pain assessments administered by nursing (Table 2). Overall, the Numeric Rating Scale was the most common tool used, comprising 68.1% of total assessments, followed by the Verbal Descriptor Tool (23.7%), the Checklist of Nonverbal Pain Indicators (7.2%), and the FACES pain scale (0.8%). Patients who received numeric ratings were more likely to be white, English speaking, younger, on opioids prior to admission, and have a lower comorbidity index. Patients receiving verbal assessments were more likely to be Asian and the least likely to be on opioids prior to admission. Patients who received the FACES pain scale and the Checklist of Non-Verbal Pain Indicators had similar baseline characteristics to each other. Compared to patients receiving other pain assessment tools, these patients were less likely to be white, and had the highest proportion with limited English proficiency, median age, and comorbidity index.

We then examined the variation in pain assessments, restricted to nursing performed “self-reported assessments,” comparing numeric ratings to either the Verbal Descriptor Tool or FACES (Supplemental Table 2). This restricted dataset comprised of 50,865 patient hospitalizations with 1,722,304 patient-level pain assessment values. Patients receiving numeric pain assessments were significantly more likely to be white (48.5% vs. 42.4%) and less likely to be Asian (12.4% vs 23.4%) than those receiving verbal/FACES assessments. Patients receiving numeric assessments compared to those receiving verbal/FACES assessments were also significantly more likely to be English speaking (91.2% vs. 79.5%), younger (55 vs. 65 median age), have a lower median comorbidity index (7 vs. 11), and to be on opioids prior to admission (58.4% vs. 41.0%).

Using multivariable logistic regression to model the relationship between race/ethnicity, LEP status, and the type of self-report tool used, compared to white patients, we found that nurses were less likely to measure pain using numeric rating tools for Asian patients (OR 0.74, 95% CI 0.70–0.78). Latino, Multi-Racial, and patients classified as Other also had significantly lower odds of receiving a numeric pain assessment compared to white patients. American Indian or Alaska Native, Black, and Native Hawaiian or Other Pacific Islander patients did not have significantly different odds compared to white patients of receiving a numeric assessment. Patients with LEP were less likely to receive a numeric assessment compared to English speaking patients (OR 0.61, 95% CI 0.58–0.65). (Table 3) A separate regression including the interaction between race/ethnicity and LEP status did not yield significantly different results (data not shown).

### Outcome 2: Opioid Medication Administration Related to Pain Assessment

MMEs for patients varied significantly across the different types of documented pain assessments (Table 4). Overall, patients with numeric ratings had the highest average daily MMEs (173.5) with verbal descriptor assessments having the lowest average (74.2). There was significant variation across racial, ethnic, and language groups within each pain assessment tool category. Within each pain assessment category, Asian and Native Hawaiian/Other Pacific Islander patients received the fewest MMEs. Black patients received the most MMEs with numeric ratings. English speaking patients received substantially more MMEs compared to patients with LEP across all pain assessments. When focusing upon nursing performed self-report assessments only, there was a similar pattern to the overall data, where Asian and Native Hawaiian/Other Pacific Islander patients, and patients with LEP, received the fewest MMEs across pain assessment categories (Supplemental Table 3).

## Discussion

This study explores the distribution of pain assessment measures for a racially and linguistically diverse patient population over several years and considers the association between the nurse’s documented pain assessment tool with actual opioid medication administered. There was significant variation in nursing performed pain assessment and opioid medication administered across baseline patient characteristics, including variation by race, ethnicity, and language status. There is a paucity of published literature regarding the differential use of pain assessment tools in adult clinical medicine by race, ethnicity, and language. Our findings that report the frequency and associated odds of different types of pain assessment tools in clinical practice across patient sociodemographic factors is novel for the fields of pain assessment, nursing, and general medicine.

Examining racial and ethnic differences, we found that Asian, Latino, Multi-Racial, and patients classified as Other had fewer odds of receiving a numeric rating compared to white patients. In terms of the association between pain assessment and opioids administered, Black patients received more opioids and Asian and Latino patients received fewer opioids than white patients. These racial disparities have basis in the literature (Becker et al., 2011; Dequeker et al., 2018; Ezenwa & Fleming, 2012; Maina et al., 2018; Morrison et al., 2021). There is probable interpersonal bias from nurses in assessing pain and clinicians in prescribing medication. Prior studies have demonstrated that nurses are less likely to document pain for Black and Latino patients, often underrate patients’ beliefs about pain management, and use demographic cues for a significant portion of pain-related decisions (Dequeker et al., 2018; Morrison et al., 2021). A recent review of the clinician bias literature found anti-minoritized group/pro-white bias in 31 of 37 published studies (Maina et al., 2018). Many clinicians are less likely to prescribe opioids because of concerns for overdose or misuse, with greater concerns by clinicians regarding non-white patients (Becker et al., 2011). While we found greater equity in pain assessment for Black patients compared to prior studies, there were significant differences in opioid medication administration across racial and ethnic groups, consistent with previous findings (Ezenwa & Fleming, 2012).

Examining differences across language status, we found that patients with LEP were less likely to receive a numeric pain rating. Across all pain assessment types, patients with LEP received fewer opioids than English speaking patients in unadjusted and adjusted analyses. Prior studies have identified the undertreatment of pain in patients with LEP (Chiauzzi et al., 2011; Schwartz et al., 2021; Silva et al., 2016). Cultural competence and language concordance between the clinician and patient promote appropriate pain management (Diamond et al., 2019). Inadequate interpreter use can lead to inadequate pain control (Jimenez et al., 2012). Evidenced by the higher use of behavioral tools for patients with LEP, it is probable that interpreters were underutilized by nurses. For example, internal data from our institution during a one-month period in 2018 indicated that video interpreters were used for less than 30% of hospital days for LEP patients (Eniasivam, Malevanchik, Khoong, Lau, & Fernández, 2020). Interestingly, in our study, even for self-reported assessments, nurses still underutilized the numeric scale. It is probable that the numeric scale is more onerous for nurses to use as it may require more explanation and time used with the interpreter.

Finally, we note the significant interplay between race, ethnicity, and LEP status. Limited English proficiency is specific to certain patient groups in our cohort. Black (99.4%), American Indian or Alaska Native (98.0%), and Latino patients (70.6%) were mostly English speaking. Alternatively, 45.5% of Asian patients had LEP. The group in our cohort with the lowest proportion of numeric ratings and the fewest daily opioids were Asian patients with LEP. Individual factors including prayer, cultural and language-based specific coping strategies and communication styles, along with provider-level discretion and bias, were all potential contributors to these differences (Meints et al., 2016, 2019). Even with proper interpreter use, there are cultural differences in how pain is conveyed, and these language differences vary within each given community (Miller & Abu-Alhaija, 2019; Sherwood et al., 2003).

### Limitations

This study had several limitations. First, the data collection for pain assessment was limited by the EHR. We only utilized complete cases where a Pain Assessment Tool was linked with a specific value. Nurses often switched between different types of pain assessment tools, meaning many patients had more than one different type of assessment. We attempted to address this by being data-inclusive, providing all the available and complete assessments for each patient. Furthermore, documentation of a patient’s race is supposed to be completed by asking the patient, but audits have demonstrated discrepancies between what is documented in the medical record and what is self-reported (Bergdall et al., 2012). Second, we did not have access to interpreter usage data. It would have been helpful to characterize the association between interpreter use and pain assessment type. Moreover, we did not have access to the identity of the nurse performing the pain assessment. Third, there is ongoing debate about the utility and comparability across patient groups of pain scales. There are significant cultural differences in how pain is communicated. While numeric scores are preferred in United States-based clinical research, separate studies of minoritized populations and patients with lower literacy may demonstrate preference for alternative pain assessment tools (Li et al., 2007; Pathak et al., 2018; Safikhani et al., 2017; Yazici Sayin & Akyolcu, 2014). There is an increased focus on the use of functional pain assessment tools, but this has not become prevalent enough to study using this dataset (Adeboye et al., 2021). At our institution, the Numeric Rating Scale is still emphasized as the standard pain assessment tool.

## Conclusions

Disparities in pain assessment and management arise from the intersection of patient (i.e. treatment and cultural preferences), clinician (i.e. assessment tools and bias), and system-wide factors (i.e. structural racism and access) (Meghani et al., 2012; Meints et al., 2016). Future studies are essential to delineate these disparities and address these shortcomings. As next steps, we plan to examine the granular relationship between individual pain assessments and subsequent medication administration. We also plan to examine provider level variation (by prescribing doctor and nurse) in pain assessment and management. Our institution is moving towards a more holistic approach to pain assessment that includes functional measures, and we hope to evaluate disparities in implementation. It remains unclear how well these various assessment tools, and quantitative data, capture a patient’s true experience of pain. Researchers should investigate patients’ qualitative experience of pain and satisfaction with care from clinicians. We must work to determine which approaches/tools to pain assessment align best with a given patient’s cultural background, communication methods, and personal treatment preferences. Specifically, the major disparities for Asian patients with LEP require specific analyses, including patient surveys, and prospective data collection.

Our findings are novel in describing pain assessment in clinical practice and highlight the continued importance of standardization in clinical care by prioritizing the use of accepted pain assessment tools, interpreters with *every* clinical encounter, and data collection through an equity lens. The identification and characterization of what inequities exist across different patient-level factors can offer direction for targeted quality improvement initiatives, including more equitable prescribing practices.

## Supplementary Material

Supplementary Material

## Figures and Tables

**Table 1:** Description of Pain Assessment Tools.

Pain Assessment Tool^a^	Type	Scale	Assessment
Numeric Rating Scale (NRS)	Self-Report	0–10, eyes closed/patient calm	Clinician asks patient “On a scale of 0–10 where 0 is no pain and 10 is the worst pain you’ve experienced, at this moment, what number represents your overall pain level?”
Verbal Descriptor Scale (VDS)	Self-Report	none, mild, moderate, severe, eyes closed/patient calm	Clinician asks patients to rate their pain using these descriptors
FACES Pain Scale Revised (FPS-R)	Self-Report	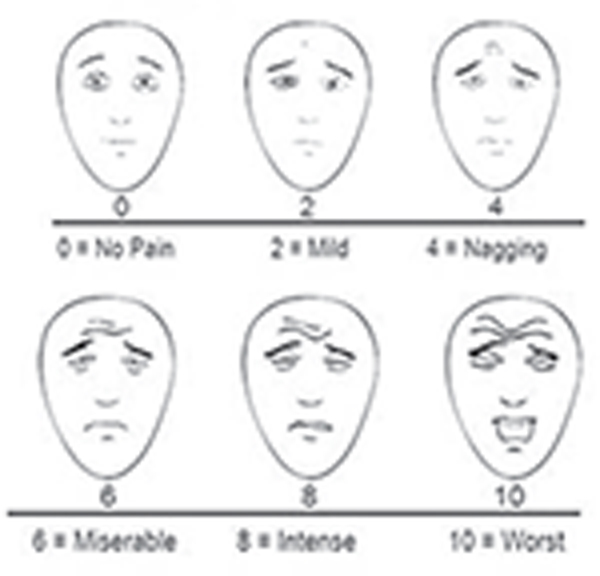	Clinician shows the patient the faces and state, “These faces show how much something can hurt.” Point to the zero picture and state, “This shows no pain.” Point to the 10 picture and state, “this one shows the worst pain experienced.” Then ask the patient, “What face best represents your pain level now?”
Checklist of Nonverbal Pain Indicators (CNPI)	Behavioral Tool	Patients are assessed for the following 1. Vocal complaints, nonverbal 2. Facial grimaces/winces 3. Bracing 4. Restlessness 5. Rubbing 6. Vocal complaints, verbal	The clinician observes the patient for one minute at rest and one minute during movement and the scores are added up
Critical Care Pain Observation Tool (CCPOT)	Behavioral Tool	Facial Expressions, Body Movements, Compliance with ventilator, vocalization, muscle tension	Observe the patient for a minute before selecting a score for each behavior. Used in the Intensive Care Setting.

**Table 2:** Baseline Patient and Clinical Characteristics of 1,858,441 Patient-level Pain Assessments, n(%)^a^.

	All Assessments	Self-Report Tool			Behavioral Tool	Other	
					
	*Overall*	*Numeric*	*Verbal*	*FACES*	*CNPI*	*Other*	*p-value*

**Total**	1,858,441	1,265,662 (68.1)	440,742 (23.7)	15,806 (0.8)	133,054 (7.2)	3,083(0.2)	<.001
**RACE/ETHNICITY**							
American Indian or Alaska Native	11,594 (0.6)	9,215 (0.7)	1,920 (0.4)	55 (0.4)	387 (0.3)	17 (0.6)	<.001
Asian	305,953 (16.5)	156,588 (12.4)	101,775 (23.1)	5,084 (32.2)	41,738 (31.4)	768 (24.9)	
Black or African American	322,474 (17.4)	237,556 (18.8)	64,269 (14.6)	2,010 (12.7)	18,187 (13.7)	452 (14.7)	
Latino	245,012 (13.2)	171,757 (13.6)	56,430 (12.8)	1,832 (11.6)	14,576 (11.0)	417 (13.5)	
Multi-Race/Ethnicity	37,853 (2.0)	25,488 (2.0)	9,325 (2.1)	296 (1.9)	2,685 (2.0)	59 (1.9)	
Native Hawaiian or Other Pacific Islander	15,024 (0.8)	8,251 (0.7)	3,813 (0.9)	186 (1.2)	2,726 (2.1)	48 (1.6)	
Other/Unknown	64,616 (3.5)	43,610 (3.5)	15,291 (3.5)	543 (3.5)	4,952 (3.7)	110 (3.6)	
White	855,915 (46.1)	613,197 (48.5)	187,908 (42.6)	5,795 (36.7)	47,803 (35.9)	1,212 (39.3)	
**GENDER**							
Female	949,950 (51.1)	649,443 (51.3)	217,858 (49.4)	8,691 (55.0)	72,382 (54.4)	1,576 (51.1)	<.001
Male	907,774 (48.9)	615,726 (48.7)	222,787 (50.6)	7,106 (45.0)	60,648 (45.6)	1,507 (48.9)	
Nonbinary	524 (0.0)	437 (0.0)	66 (0.0)	2 (0.0)	19 (0.0)	0 (0.0)	
Unknown	193 (0.0)	150 (0.0)	31(0.0)	7 (0.0)	5 (0.0)	0 (0.0)	
**LIMITED ENGLISH PROFICIENCY STATUS**							
Yes	244,794 (13.2)	111,700 (8.8)	88,758 (20.1)	4,766 (30.2)	38,844 (29.2)	726 (23.6)	<.001
No	1,613,647 (86.8)	1,154,056 (91.2)	351,984 (79.9)	11,040 (69.9)	94,210 (70.8)	2,357 (76.5)	
**OTHER COVARIATES**							
Age, median (IQR)	59 (44–71)	55 (40–67)	65 (52–77)	69 (54–83)	69 (54–84)	65 (52–81)	<.001
Cancer Pain Dx	44,100 (2.4)	30,714 (2.4)	8,713 (2.0)	369 (2.3)	4,202 (3.2)	102 (3.3)	<.001
Opioids on Admission	984,914 (53.0)	739,465 (58.4)	180,365 (40.9)	6,647 (42.1)	56,838 (42.7)	1,419 (46.0)	<.001
Comfort Care	101,320 (5.5)	38,239 (3.0)	25,552 (5.8)	1,828 (11.6)	35,142 (26.4)	509 (16.5)	<.001
Comorbidity Index, median (IQR)	9 (0–17)	7 (0–15)	11 (2–19)	11 (2–20)	13 (4–21)	10 (1–19)	<.001
LOS, median (IQR)	7.8 (4.2–15.2)	7.6 (4.1–15.0)	7.8 (4.5–15.1)	8.3 (4.6–16.8)	8.8 (4.8–16.7)	6.1 (3.5–12.4)	<.001

a The percentages reflect column percentages.

**Table 3:** Likelihood of Having a Numeric Pain Tool Used When Assessing Pain Among 51,602 Patient Hospitalizations (Results from the Multivariate Model).

	Adjusted Odds Ratio, 95% CI	*P* value

**RACE/ETHNICITY**		
American Indian or Alaska Native	1.21 (0.99–1.49)	.068
Asian	0.74 (0.70–0.78)	<.001
Black or African American	0.98 (0.90–1.05)	.545
Latino	0.93 (0.87–1.00)	.039
Multi-Race/Ethnicity	0.84 (0.75–0.94)	.002
Native Hawaiian or Other Pacific Islander	0.88 (0.74–1.06)	.177
Other/Unknown	0.81 (0.73–0.91)	<.001
White	ref	-
**GENDER**		
Female	ref	-
Male	0.98 (0.94–1.02)	.253
Nonbinary	2.28 (1.54–3.38)	<.001
Unknown	1.32 (0.23–7.70)	.757
**LIMITED ENGLISH PROFICIENCY STATUS**		
Yes	0.61 (0.58–0.65)	<.001
No	ref	-
**OTHER COVARIATES**		
Time (by year of study)	0.82 (0.81–0.83)	<.001
Age (year)	0.98 (0.98–0.98)	<.001
Cancer Pain	1.38 (1.21–1.58)	<.001
Comfort Care	0.55 (0.50–0.60)	<.001
Opioid on Admission	1.62 (1.56–1.69)	<.001
Length of Stay (day)	1.00 (1.00–1.00)	<.001
Comorbidity Index (Elixhauser Score)	0.99 (0.99–0.99)	<.001

**Table 4:** Average Daily Morphine Milligram Equivalent (MME) by Pain Assessment Type Across 1,858,441 Patient-level Pain Assessments.

	Numeric	Verbal	FACES	CNPI	Other	*p* value

Overall	173.5	74.2	129.3	242.6	202.4	<.001
**RACE/ETHNICITY**						
American Indian or Alaska Native	138.2	83.8	92.3	115.8	57.8	<.001
Asian	75.7	37.8	55.6	79.8	104.8	
Black or African American	239.8	79.4	115.1	137.7	158.9	
Latino	142.0	56.7	116.5	246.2	182.6	
Multi-Race/Ethnicity	177.3	208.2	75.8	2522.8	416.8	
Native Hawaiian or Other Pacific Islander	57.4	35.0	23.2	1654.5	1092.2	
Other/Unknown	159.5	44.8	43.9	102.9	152.1	
White	184.4	93.9	217.4	230.5	248.1	
**LIMITED ENGLISH PROFICIENCY**						
Yes	46.5	20.7	34.6	48.6	35.6	<.001
No	185.7	87.7	170.2	322.7	253.7	
