# Supplementary material for: Pain Assessment Disparities by Race, Ethnicity, and Language in Adult Hospitalized Patients
Source: Pain Manag Nurs. Author manuscript; Available in PMC 2024 Mar 20. (PMC10954313; doi:10.1016/j.pmn.2023.03.012)
Supplement: Supplementary Material [file NIHMS1970658-supplement-Supplementary_Material.docx]

**Supplemental Table 1: Baseline patient and clinical characteristics of 51,602 patients included in this study, n (%)**

| **RACE/ETHNICITY** | |
| --- | --- |
| American Indian or Alaska Native | 230 (0.45%) |
| Asian | 10,796 (20.9%) |
| Black or African American | 7,650 (14.8%) |
| Latino | 6,112 (11.8%) |
| Multi-Race/Ethnicity | 1,106 (2.1%) |
| Native Hawaiian or Other Pacific Islander | 520 (1.0%) |
| Other/Unknown | 1,731 (3.4%) |
| White | 23,457 (45.5%) |
| **LIMITED ENGLISH PROFICIENCY STATUS** | |
| Yes | 8,404 (16.3%) |
| No | 43,198 (83.7%) |
| **GENDER** | |
| Male | 25,646 (49.7%) |
| Female | 25,943 (50.3%) |
| Non-Binary | 9 (0.0%) |
| Unknown | 4 (0.0%) |
| **OTHER COVARIATES** | |
| Age, mean | 60.8 years |
| Cancer Pain Dx | 4.6% |
| Opioids on Admission | 42.1% |
| Comorbidity Index, mean | 8.2 |
| Length of Stay, mean | 5.35 days |

| **Supplemental Table 2: Baseline patient and clinical characteristics of 1,722,194 patient-level self-reported pain assessments, *n* (%)^1^** | | | | |
| --- | --- | --- | --- | --- |
|  | **Numeric** | **Verbal/FACES** | | **p-value** |
| Total | 1,265,662 (73.5) | 456,532 (26.5) | |  |
| **RACE/ETHNICITY** | | | | |
| American Indian or Alaska Native | 9,215 (0.7) | 1,975 (0.4) | | <0.001 |
| Asian | 156,588 (12.4) | 106,859 (23.4) | |  |
| Black or African American | 237,556 (18.8) | 66,279 (14.5) | |  |
| Latino | 171,757 (13.6) | 58,262 (12.8) | |  |
| Multi-Race/Ethnicity | 25,488 (2.0) | 9,621 (2.1) | |  |
| Native Hawaiian or Other Pacific Islander | 8,251 (0.7) | 3,999 (0.9) | |  |
| Other/Unknown | 43,610 (3.5) | 15,834 (3.5) | |  |
| White | 613,197 (48.5) | 193,703 (42.4) | |  |
| **GENDER** | | | | |
| Female | 649,443 (51.3) | 226,549 (49.6) | | <0.001 |
| Male | 615,726 (48.6) | 229,893 (50.4) | |  |
| Non-binary | 437 (0.0) | 68 (0.0) | |  |
| Other | 56 (0.0) | 22 (0.0) | |  |
| **LIMITED ENGLISH PROFICIENCY STATUS** | | | | |
| Yes | 111,700 (8.8) | 93,524 (20.5) | | <0.001 |
| No | 1,153,962 (91.2) | 363,008 (79.5) | |  |
| **OTHER COVARIATES** | | | | |
| Age, median (IQR) | 55 (40-67) | | 65 (52-78) | <0.001 |
| Cancer Pain Dx | 30,714 (2.4) | | 9,082 (2.0) | <0.001 |
| Opioids on Admission | 739,645 (58.4) | | 187,012 (41.0) | <0.001 |
| Comfort Care | 38,289 (3.0) | | 27,380 (6.0) | <0.001 |
| Comorbidity Index, median (IQR) | 7 (0-15) | | 11 (2-19) | <0.001 |
| LOS, median (IQR) | 7.6 (4.1-15.0) | | 7.8 (4.5-15.2) | <0.001 |

^1^ the percentages reflect column percentages

| **Supplemental Table 3: Average daily morphine milligram equivalent (MME) across 1,722,194 patient-level self-reported pain assessments** | | | |
| --- | --- | --- | --- |
|  | **Numeric** | **Verbal/FACES** | **p-value** |
| **Overall** | 173.5 | 76.1 | <0.001 |
| **Race/Ethnicity** | | | |
| American Indian or Alaska Native | 138.2 | 84.0 | <0.001 |
| Asian | 75.7 | 38.6 |  |
| Black or African American | 239.8 | 80.5 |  |
| Latino | 142.0 | 58.6 |  |
| Multi-Race/Ethnicity | 177.3 | 204.1 |  |
| Native Hawaiian or Other Pacific Islander | 57.4 | 34.4 |  |
| Other/Unknown | 159.5 | 44.8 |  |
| White | 184.4 | 97.6 |  |
| **Limited English Proficiency Status** | | | |
| Yes | 46.5 | 21.4 | <0.001 |
| No | 185.7 | 90.2 |  |
